# Stroke Mimic in a Cancer Survivor: A Rare Case of SMART (Stroke-Like Migraine Attacks After Radiation Therapy) Syndrome

**DOI:** 10.7759/cureus.92642

**Published:** 2025-09-18

**Authors:** Radhika Saigal, Edwin Mazhuppel Rechard, Lokesh Koumar Sivanandam

**Affiliations:** 1 Internal Medicine, Walsall Healthcare NHS Trust, Walsall, GBR; 2 Internal Medicine, The Royal Wolverhampton NHS Trust, Wolverhampton, GBR; 3 Internal Medicine, Torbay and South Devon NHS Foundation Trust, Torquay, GBR

**Keywords:** migraine, post radiation effect, radiation oncology complication, smart syndrome, stroke mimic

## Abstract

Stroke-like migraine attacks after radiation therapy (SMART) syndrome is a rare and delayed side effect of cranial irradiation. It can appear after many years following radiation therapy and is characterised by temporary, stroke-like neurological deficits such as hemiparesis, seizures, aphasia, sensory abnormalities, and headaches. Although the condition is self-limiting, it can pose a difficulty in diagnosis due to its symptoms coinciding with other neurological disorders.

We report a case of a 63-year-old male with a diagnosis of oligodendroglioma for which he had previously received cranial irradiation in 2015. He presented to the emergency room in 2025, 10 years later, with an abrupt-onset weakness in his left upper and lower limbs. Acute causes like infection, stroke, and seizures were ruled out through investigations. The patient’s history, clinical presentation, and radiological exclusion of other pathologies led to the diagnosis of SMART syndrome. Verapamil was prescribed as migraine prophylaxis, and neurology follow-up was part of the treatment plan. At the subsequent follow-up, it was reported that the clinical condition was showing gradual improvement.

Patients with a history of cranial irradiation and temporary focal neurological symptoms should be evaluated for SMART syndrome, especially when other acute pathologies are excluded, as early detection can help avoid needless interventions and guide appropriate management.

## Introduction

Stroke-like migraine attacks after radiation therapy (SMART) syndrome is an uncommon and delayed side effect of cranial irradiation [1-3]. Its clinical and radiological overlap with more prevalent and severe neurological conditions, such as stroke, seizures, recurrent tumours, and central nervous system (CNS) infections, makes its clinical diagnosis challenging [3-4]. SMART syndrome was first described in 1995, and since then, there have been fewer than 100 cases reported in the literature [2-3]. However, the real incidence of SMART syndrome may be much higher due to the advances in modern medicine as well as the increase in cancer survival rates and the rise of cranial radiotherapy worldwide, necessitating a greater clinical awareness of SMART syndrome [5-7].

SMART syndrome is clinically associated with temporary episodic neurological symptoms, which include hemiparesis, headaches, aphasia, visual field deficits, seizures, and sensory disturbances [1-3]. These symptoms frequently call for immediate investigations as they can resemble an acute stroke or complex migraine. Usually occurring in patients treated for primary brain tumours like gliomas, medulloblastomas, or metastases, the syndrome appears years after the end of radiation therapy. It is more common in middle-aged and older adults who have had longer survival times following cancer treatment and is most frequently linked to high-dose radiation therapy, which usually exceeds 50 Gy [2].

The pathophysiology of SMART syndrome is still not fully understood; however, cortical spreading depression, impaired cerebral autoregulation, delayed radiation-induced endothelial damage, and blood-brain barrier disruption are some of the hypothesised mechanisms [2-3]. The observed stroke-like symptoms could be caused by temporary cortical dysfunction in the previously exposed brain areas because of these alterations [2]. Neuroimaging can aid in the diagnosis with classic findings of unilateral cortical thickening, T2/FLAIR hyperintensities, and transient gadolinium-enhancing lesions limited to previously irradiated regions. MRI is a key diagnostic tool [2,3,6,7]. However, imaging may be normal in certain situations, necessitating the combination of clinical history and the exclusion of alternative possible causes.

The treatment options for SMART syndrome are mainly prophylactic. Corticosteroids and migraine-preventive medications like calcium channel blockers or antiepileptics are among the main treatment options [2,7]. This case report of SMART syndrome involves a patient who underwent cranial irradiation for oligodendroglioma 10 years ago in 2015. We describe the clinical course, diagnostic considerations, management approaches, and follow-up care for this unique but significant neurological entity.

## Case presentation

A 63-year-old male with a diagnosis of oligodendroglioma grade three in the right parietal region with 1p/19q co-deletion underwent cranial irradiation therapy in 2015. He received 54 Gy of cranial radiation in 30 fractions, which was followed by five adjuvant cycles of PCV (procarbazine, lomustine, and vincristine) chemotherapy. In 2025, the patient presented to the emergency department with sudden-onset weakness in his left upper and lower limbs, accompanied by mild confusion and a diffuse throbbing headache, but without paraesthesia, nausea, vomiting, visual changes, or seizure activity. On examination, his Glasgow Coma Scale was 11 (E3 M4 V4). Neurological assessment revealed hypotonia with power 2/5 and diminished sensation in the left upper limb, hypotonia with power 3/5 and preserved sensation in the left lower limb, while motor and sensory function on the right side was normal; systemic examinations were unremarkable.

A non-contrast CT head was arranged for this patient, which was negative for any acute intracranial pathology. After discussion with the stroke team, inpatient admission for further evaluation was recommended. The medical team began empirical treatment with intravenous ceftriaxone for suspected meningitis. However, lumbar puncture cerebrospinal fluid (CSF) analysis was later found to be unremarkable. The blood panel for inflammatory markers and electrolytes were all within normal ranges.

**Figure 1:**
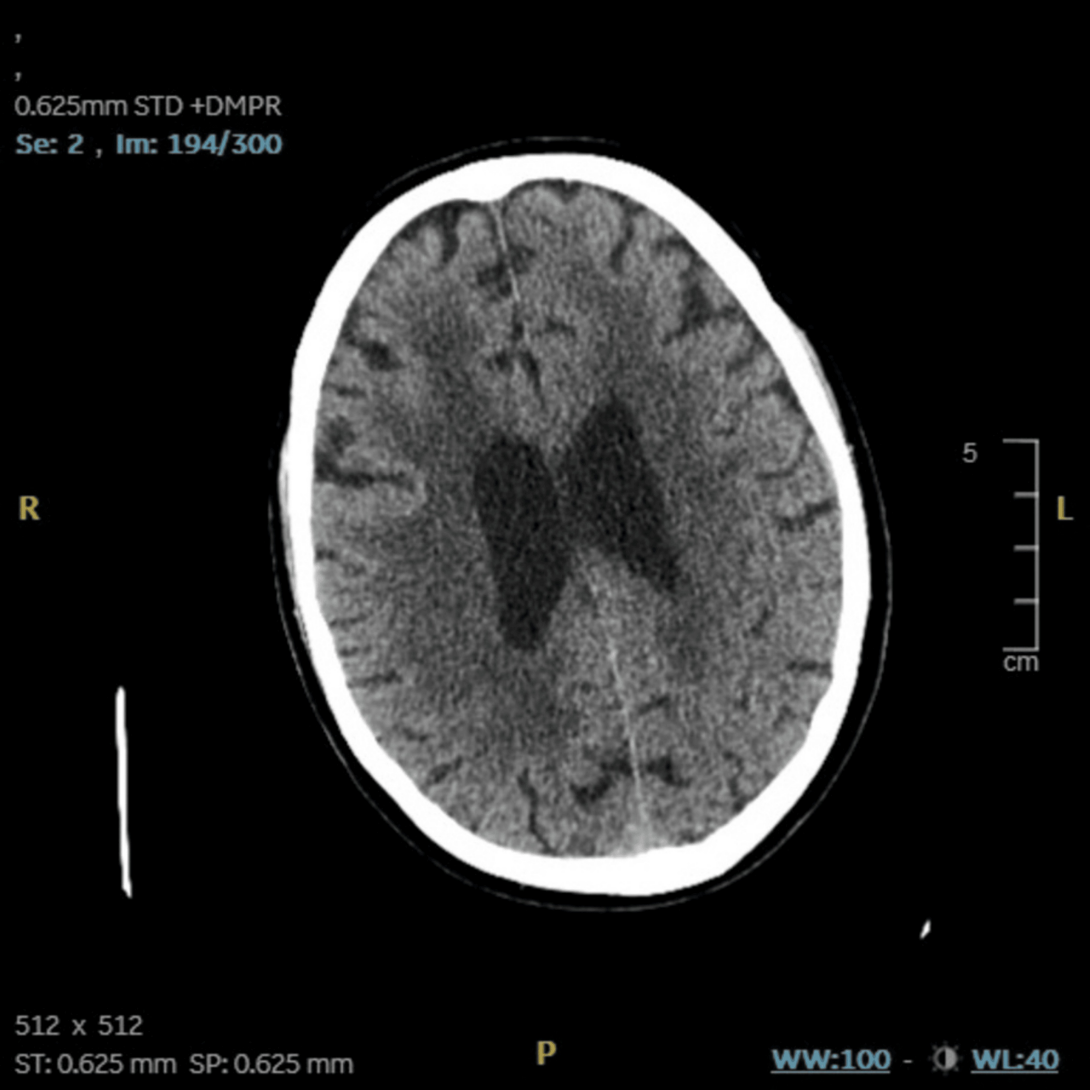
CT head - image 1 The image shows patchy areas of hypoattenuation in the cerebral white matter, consistent with moderate small vessel disease CT: computed tomography

**Figure 2:**
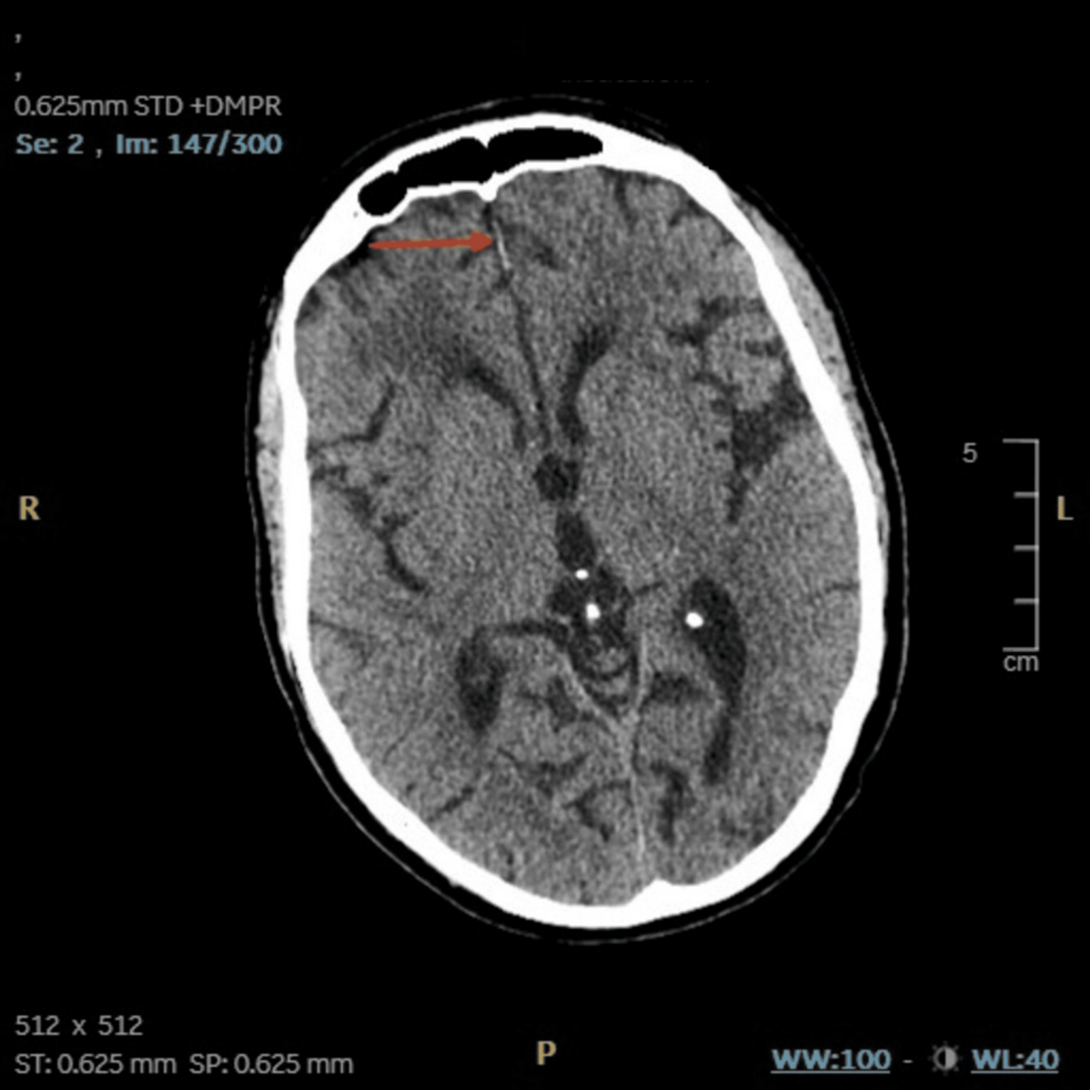
CT head - image 2 The red arrow signifies fine specks of calcification in the right corona radiata and right high frontal region CT: computed tomography

**Figure 3:**
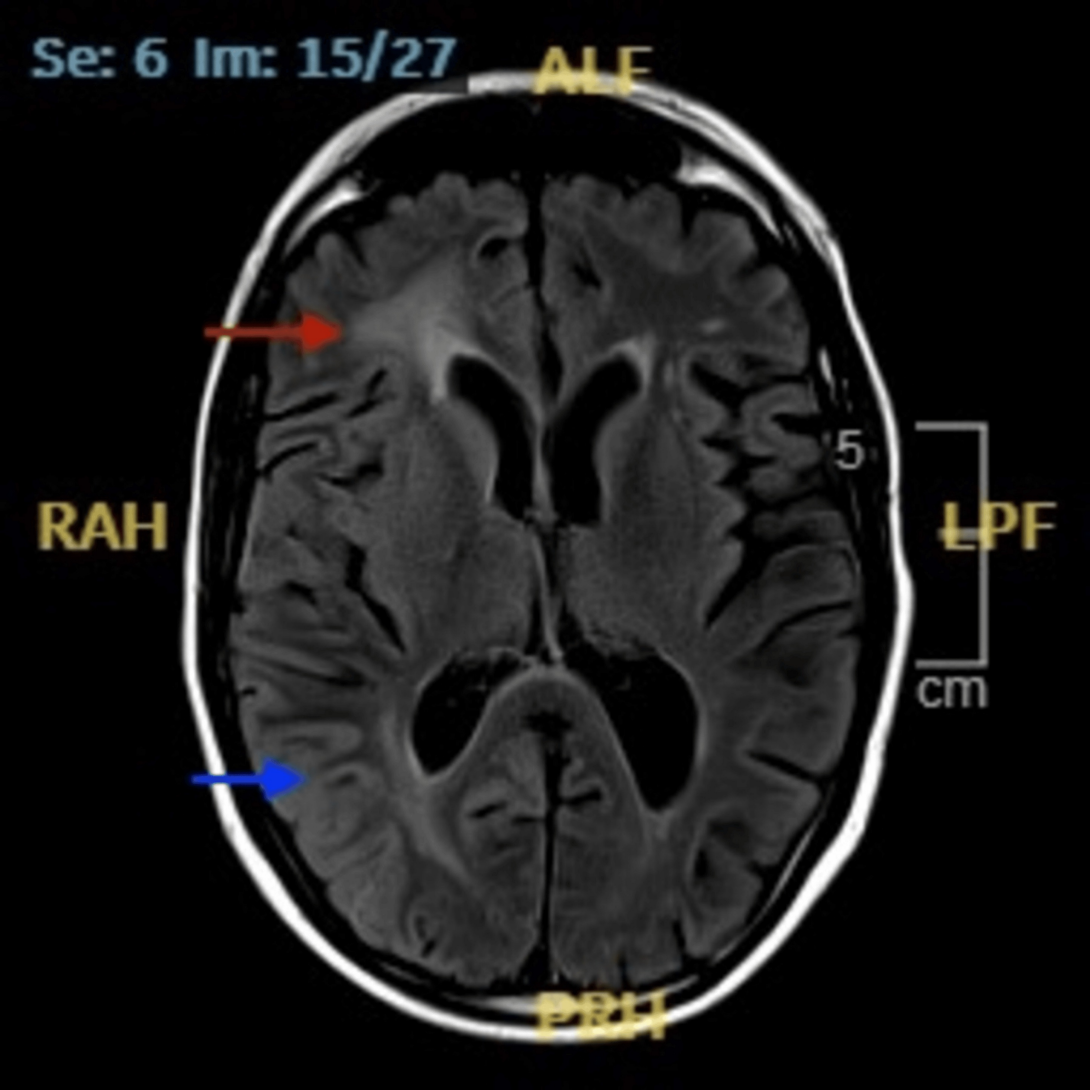
MRI brain (T2/FLAIR) Red arrow: small vessel ischemic changes. Blue arrow: mild gyral enhancement as compared to the left side MRI: magnetic resonance imaging; FLAIR: fluid attenuated inversion recovery

The ongoing confusion and motor impairments, along with the unremarkable investigation findings from the CSF and blood panel, necessitated input from the neurology team. The non-contrast CT showed patchy areas of hypoattenuation in the cerebral white matter (Figure 1) and specks of calcification in the right corona radiata and right high frontal region (Figure 2). On gathering further information from the patient and next of kin, it was revealed that the patient had experienced two previous episodes with similar symptoms in the last six months, including temporary confusion and left-sided weakness, both of which had resolved on their own. He had been started on levetiracetam (1000 mg morning, 500 mg evening) for a suspected focal seizure during one of these episodes. MRI brain revealed marked peri-ventricular white matter changes in the superior periventricular regions, more marked on the right with minimal gyral enhancement (Figure 3). These findings were reported to be likely a consequence of radiotherapy and age-related small vessel disease.

For further investigations, an EEG was performed as per neurology advice, which revealed no abnormalities. SMART syndrome was therefore diagnosed, given the combination of the patient's history with cranial irradiation, subtle radiological findings, normal EEG, and recurrent, temporary neurological symptoms. Migraine prophylactic therapy of oral verapamil 40 mg twice a day was initiated with physiotherapy and scheduled neurology follow-up. The patient later reported improvement in motor function (power increased to 3/5 in the upper limb and 4/5 in the lower limb) and headache intensity at the follow-up.

## Discussion

SMART syndrome is a rare but possible side effect of cranial irradiation and is typically identified by exclusion or retrospective diagnosis [1,3,4]. However, as the symptoms of SMART syndrome are non-specific and temporary, it can be challenging to diagnose without careful clinical attentiveness to patient history, as well as differentials for the symptoms such as ischaemic stroke, seizure activity, recurrent malignancy, and CNS infection [6]. In our patient, SMART syndrome was diagnosed almost ten years after undergoing cranial radiation therapy for oligodendroglioma. The diagnosis of SMART syndrome was established through exclusion, which was supported by strong clinical presentation, subtle radiological findings, normal CSF, normal EEG, and exclusion of alternative differentials (stroke, seizure, tumour recurrence). 

The preferred modality for the diagnosis of SMART syndrome is MRI imaging [2,4,6]. In previously irradiated areas, it usually shows gyral enhancement, unilateral cortical swelling, and T2/FLAIR hyperintensities [4,6,7,8]. However, MRI findings for this case highlighted the subtle T2 white matter hyperintensities along with minimal gyral enhancement corresponding to the previously irradiated areas (Figure 3). Comparable cases with subtle or negative imaging have been reported, though they remain relatively rare in the literature. 

The treatment for SMART syndrome primarily involves supportive care and symptom management [5]. Corticosteroids have been used in some instances with variable outcomes, while calcium channel blockers such as verapamil have shown some efficacy in preventing migraine-like symptoms [2,7]. In our patient, verapamil was well tolerated and was associated with both symptomatic and functional improvement. Additionally, the choice of verapamil over corticosteroids also helped avoid the potential adverse effects of long-term steroid therapy.

Patients with SMART syndrome are susceptible to recurrence even though they may fully recover. Close monitoring is therefore advised, particularly in cases where symptoms are progressive or recurrent. On successive follow-ups, our patient reported significant symptomatic improvement. This report highlights two key observations: (1) SMART syndrome can occur many years after cranial radiation; and (2) its diagnosis should not be excluded in the absence of typical MRI findings, provided the clinical history and symptom profile strongly support the condition.

## Conclusions

SMART syndrome is a rare complication of cranial irradiation that manifests as temporary neurological deficits that resemble migraine or stroke. A high index of suspicion is necessary for its diagnosis, especially in patients with a history of previous brain radiation therapy and who have recurrent focal neurological symptoms. This report emphasises the importance of considering SMART syndrome in the differential diagnosis when more conventional investigations yield no definitive results. Patient care can be enhanced with early recognition and with the delivery of appropriate management and the avoidance of unnecessary investigations. Awareness of SMART syndrome among physicians is essential, as the incidence of such clinical presentations is possibly on the rise due to improved cancer survival rates and cranial radiotherapy worldwide.
